# Breathing exercise for hypertensive patients: A scoping review

**DOI:** 10.3389/fphys.2023.1048338

**Published:** 2023-01-25

**Authors:** Isnaini Herawati, Arimi Fitri Mat Ludin, Mutalazimah M, Ismarulyusda Ishak, Nor M. F. Farah

**Affiliations:** ^1^ Biomedical Science Programme & Center for Healthy Ageing and Wellness (HCARE), Faculty of Health Sciences, Universiti Kebangsaan Malaysia, Jalan Raja Muda Abdul Aziz, Kuala Lumpur, Malaysia; ^2^ Faculty of Health Sciences, Universitas Muhammadiyah Surakarta, J. A.Yani Tromol Pos 1 Pabelan Kartasura, Surakarta, Indonesia; ^3^ Biomedical Science Programme & Center for Toxicology and Health Risk (CORE), Faculty of Health Sciences, Universiti Kebangsaan Malaysia, Jalan Raja Muda Abdul Aziz, Kuala Lumpur, Malaysia; ^4^ Occupational Therapy Programme & Center for Community Health Studies (REACH), Faculty of Health Sciences, Universiti Kebangsaan Malaysia, Jalan Raja Muda Abdul Aziz, Kuala Lumpur, Malaysia

**Keywords:** breathing exercise, slow breathing, blood pressure, hypertension, heart rate

## Abstract

**Background:** Non-pharmacological management of hypertension includes weight loss, alcohol and sodium restriction, regular exercise, and relaxation. In people with overweight hypertension, systolic blood pressure (SBP) and diastolic blood pressure (DBP) can be decreased *via* exercise and weight loss together. Breathing exercises are one method of relaxing.

**Objectives:** The aim of this scoping review is to map the information that is currently available about the advantages of breathing exercises in decreasing blood pressure in hypertension patients.

**Methods:** This scoping review adheres to Arksey and O’Malley’s framework, which entails identifying review questions, seeking pertinent evidence, choosing pertinent studies, mapping data, and discussing, concluding, and reporting the findings. The PRISMA flowchart is used to show how the evidence search process works.

**Results:** As a result, 339 articles in total were retrieved from the three databases. 20 papers total were included in this review after screening. In 14 of the 20 investigations, participants with stage 1 and stage 2 essential hypertension, two with pre-hypertension, and four with Isolated Systolic Hypertension (ISH) were studied. The respondents’ ages ranged from 18 to 75. The systolic blood pressure declined by 4–54.22 mmHg, while the diastolic blood pressure dropped by 3–17 mmHg.

**Conclusion:** Slow breathing can be used as an alternate, non-pharmacological therapy for hypertension individuals to reduce blood pressure.

**Systematic Review Registration:** (https://osf.io/ta9u6/).

## 1 Introduction

High blood pressure, often known as hypertension, is a serious medical condition that increases the risk of heart disease, stroke, kidney failure, and other disorders. In the past 30 years, the number of adults aged 30–79 years with hypertension has risen from 650 million to 1.28 billion, with approximately half of these people unaware of their condition. According to WHO data from 2018, around 26.4 percent of the world’s population has hypertension, with a male-to-female ratio of 26.6 percent and 26.1 percent, and approximately 60 percent of those with hypertension live in developing countries, including Indonesia. In 2018, the National Institute of Basic Health Research (Riskesdas) reported an increase in the prevalence of hypertension in Indonesia’s 260 million population, which was 34.1 percent in 2018 compared to 27.8 percent in 2013 (Kementerian Kesehatan Republik Indonesia, 2019). Hypertension is also called the silent killer because it often occurs without significant complaints, so the patient does not know he has hypertension and is only known after complications occur. Symptoms usually appear after 20 years of being diagnosed with hypertension and are only known when it is affecting other organs such as the heart, kidneys, brain, and eyes. This leads to the delayed treatment and reduces life expectancy. Hypertension is frequently linked to other metabolic syndrome. In fact, one or more metabolic risk factors are present in more than 80% of hypertension patients. This clinical illness is becoming more common worldwide, and it is obviously linked to modern lifestyles marked by a lack of physical activity, resulting in overweight or obesity (Neves et al., 2013).

Pharmacological management of hypertension are often used to lower morbidity and mortality among adults with hypertension and prehypertension, by achieving and maintaining arterial blood pressure at or less than 140/90 mmHg. Keeping blood pressure under control can lower the chance of developing cardiovascular disease (Sierra & de la Sierra, 2008). Evidence suggests that pharmacological therapy for high blood pressure has limitations in regulating the condition and avoiding side effects (O’Brien, 2017). In lieu of this, individuals with hypertension should adopt a lifestyle or behavior modification strategy to lower blood pressure and prevent cardiovascular disease (Wang et al., 2010) (Wang et al., 2018) (Zhang and Moran, 2017) (Booth et al., 2017). Non-pharmacological management includes weight loss, alcohol and sodium restriction, regular exercise, and relaxation. Exercise and weight loss together have been demonstrated to reduce systolic blood pressure (SBP) and diastolic blood pressure (DBP) in overweight hypertension patients by 12.5 and 7.9 mmHg, respectively (Bacon et al., 2004).

Breathing exercise is a useful non-pharmacological interventions in controlling hypertension (Gavish, 2010). It is hypothesized that the stimulation of heart-lung mechanoreceptors during prolonged inhalation and exhalation can increase baroreflex sensitivity (BRS) and reduce sympathetic activity and chemoreflex activation (Joseph et al., 2005) (Forouzanfar et al., 2016). Deep breathing exercises at a respiratory rate of 6 or 10 breaths per minute prolongs the contraction of the diaphragm, minimize the frequency of respiration, and increase the volume of inspiration and expiration to maximize the amount of oxygen that enters the bloodstream, as well as resulting in arteriolar dilation (Ma et al., 2017). In people with hypertension, this breathing exercise can reduce SBP and DBP (Wang et al., 2010) (Chen et al., 2017). Diaphragmatic Deep Breathing has been shown to have a therapeutic effect on the physical and psychological health of people with hypertension (Ma et al., 2017) (Subbalakshmi et al., 2014) (Vasuki and Sweety, 2017) (Chen et al., 2017) (D’silva et al., 2014). Several studies have demonstrated the effect of slow breathing exercises on baroreflex sensitivity (BRS), BP, and Autonomic nervous system (ANS) function (Oneda et al., 2010) (Fonkoue et al., 2018).

Although there are available reviews on breathing exercise, however there is none specifically addressed the outcome of breathing exercise on hypertensive patients. This review would provide an overview on the effect of breathing exercise particularly on patients with hypertension. In this scoping review, we aim to map out the evidence from available studies and relevant literature regarding breathing exercises to support the management of hypertensive patients. The specific objectives of this scoping review are:

i) To summarize the research that has been done so far on the intervention method of breathing exercises for patients with hypertension.
ii) To visualize the reported outcomes (Blood pressure and heart rate) for hypertension patients who had breathing exercise intervention
iii) To determine the breathing exercise intervention’s gaps and restrictions for patients with hypertension.

## 2 Methods

**FIGURE 1:**
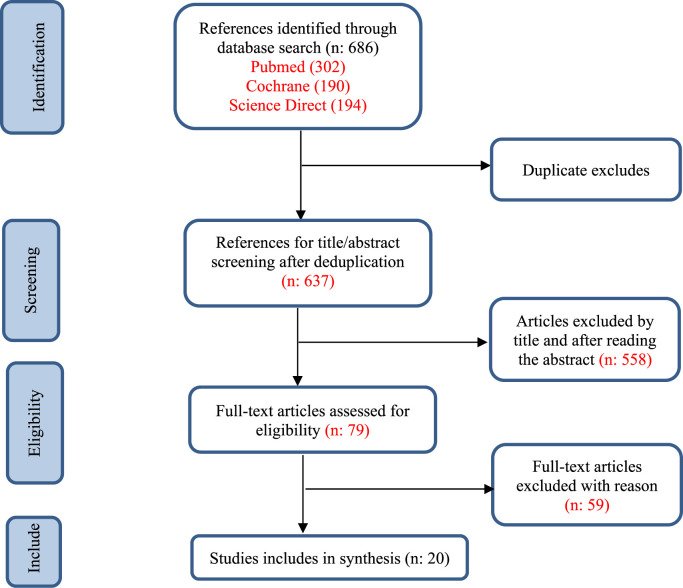
Flowchart of study selection.

This scoping review was conducted according to the PRISMA Extension for Scoping Reviews (Tricco et al., 2018), thus employing a systematic approach to mapping the evidence from available studies and relevant literature regarding breathing exercises to support the management of hypertensive patients (Figure 1). The detailed protocol for this scoping review has been registered on Open Science Foundation website (Herawati et al., 2021).

### 2.1 Inclusion criteria

The inclusion criteria for the search will be empirical and theoretical studies related to breathing exercises for hypertensive patients aged 18 and above without any other uncontrolled cardiovascular or other diseases, with or without antihypertensive medications. For those on treatment, they should be stable on anti-hypertensive treatment for a minimum of 2 months prior to the study and no change in medications during participation in the trial, published in English, in the period from August 2010 to November 2022. Studies including multiple modalities of complementary and alternative medicine (CAM) techniques and research where breathing exercises are combined with other modalities will be excluded.

### 2.2 Literature search strategy

In brief, relevant studies were retrieved from electronic databases (i.e., PubMed, COCHRANE, and Science Direct). A list of references from reviews discovered through electronic searches was checked to ensure that pertinent papers were included in the scoping review. We also looked for several sources of gray literature, such as the websites of local, national, and worldwide organizations and related scientific or medical organizations, to ensure that all pertinent material is considered.

To build a search strategy, given the research questions mentioned above, literature searches from articles were guided by PCC: Population (hypertensive patients), Concept (breathing exercise), and Content (global- no geographic target). Search terms are generated by the research team member from keywords, subject headings and synonyms such as “breathing exercise”, “hypertension”, “heart rate”, and “quality of life”, to get an overview of all potential resources from the databases. The list of keywords is shown in Table 1. The search strings are generated using the Boolean operators “AND” and “OR”, as needed. Table 2 shows the search strings generated. Each search result will be documented, and selected articles will be exported to a separate folder using the Endnote 17 reference management software.

**TABLE 1:** List of keywords and synonyms generated as search terms.

Breathing exercise	Hypertension	Heart rate	Quality of life
Breathing training	Hypertensive	Heartbeats	Life quality
Breathing technique	High blood pressure	Pulse pressure	Health-related quality of life
Breathing control			Quality of healthcare
Breathing practice			
Respiratory training			
Respiratory muscle exercise			
Diaphragmatic breathing			
Slow breathing			

**TABLE 2:** List of search strings.

Search string 1	“Breathing exercise” OR “breathing control” OR “breathing training” OR “breathing technique” OR “breathing practice” OR “slow breathing” OR “respiratory training” OR “respiratory exercise” OR “respiratory muscle training” OR “diaphragmatic breathing” AND “hypertensive” OR “hypertension” OR “high blood pressure” OR “elevated blood pressure”AND “pulse pressure” OR “heart rate” OR “autonomic nervous system”
Search string 2	“Breathing exercise” OR “breathing control” OR “breathing training” OR “breathing technique” OR “breathing practice” OR “slow breathing” OR “respiratory training” OR “respiratory exercise” OR “respiratory muscle training” OR “diaphragmatic breathing” AND “hypertensive” OR “hypertension” OR “high blood pressure” AND “quality of life” OR “life quality” OR “health-related quality of life”

### 2.3 Data extraction and charting

Screened articles that passed the initial selection process and were deemed suitable because they met the inclusion criteria—After the title and reading of the abstract were subjected to full-text analysis. From each study taken. First author’s name; Year of publication; Title; Population characteristics; Study design; The number of samples; Age and gender; and outcome. Two reviewers extracted the data, and disagreements were resolved by convention.

### 2.4 Collecting, summarizing, and reporting the result

Results from the data extraction table was collated and summarised according to our review objectives. Even though it is nota compulsory in a scoping review, we also included quality appraisal to strengthen our finding. Assessment of literature quality was conducted using PEDro quality scale, which is an 11-item scale assessing internal and external validity of clinical trials. The PEDro scale scores can range from 0 to 10, with a higher score indicating better methodological quality. Responses to items 2 to 11 are summed to create a total score, and item 1 relates to external validity Results ((Maher et al., 2003).

## 3 Results

### 3.1 Quality assessment

PEDro scores ranged from 5 to 10 points, with a mean score of 7.4 (Table 3). All of the selected studies but one (Adhana et al.) scored 6 or more, indicating the high quality of the selected trials. From the quality assessment using PEDro, we found that all study explicitly describe the eligibility criteria. Only two study [23, 38] did not employ random allocation and on top of that, [38] did not conceal participant allocation. In term of blinding, all participants and therapist were blinded only in three studies [26, 29, 33] and [18, 32, 33] accordingly. Whereby assessors were blinded in 5 of the included studies [18, 22, 26, 32, 33]. The rest of the PEDro criteria were abided by all of the included studies.

**TABLE 3:** PEDro scale quality assessment of the articles.

Reference	Eligibility criteria	Random allocation	Concealed allocation	Group similar at baseline	Blinded subjects	Blinded therapist	Blinded assessors	Less than 15% dropouts	Intention-to-treat analysis	Between-group comparisons	Point measure and variability	PEDro score
Jones et al. (2010)	yes	yes	yes	yes	yes	no	yes	yes	yes	yes	yes	9
Lin et al. (2012)	yes	yes	yes	yes	no	no	no	yes	yes	yes	yes	7
de Barros et al. (2017)	yes	yes	yes	yes	no	no	no	yes	yes	yes	yes	7
Wang et al. (2021)	yes	no	yes	yes	no	no	no	yes	yes	yes	yes	6
Hering et al. (2013)	yes	yes	yes	yes	no	no	yes	yes	yes	yes	yes	8
Wang et al. (2010)	yes	yes	yes	yes	no	no	no	yes	yes	yes	yes	7
Mitsungnern et al. (2021)	yes	yes	yes	yes	no	yes	yes	yes	yes	yes	yes	9
Anderson et al. (2010)	yes	yes	yes	yes	no	no	no	yes	yes	yes	yes	7
Sangthong et al. (2016)	yes	yes	yes	yes	no	no	no	yes	yes	yes	yes	7
Jones et al. (2015)	yes	yes	yes	yes	no	no	no	yes	yes	yes	yes	7
Ping et al. (2018)	yes	yes	yes	yes	yes	yes	yes	yes	yes	yes	yes	10
Li et al. (2018)	yes	yes	yes	yes	no	no	no	yes	yes	yes	yes	7
Ublosakka-Jones et al. (2018)	yes	yes	yes	yes	yes	no	no	yes	yes	yes	yes	8
Kalaivani et al. (2019)	yes	yes	yes	yes	no	no	no	yes	yes	yes	yes	7
de Barros et al. (2017)	yes	yes	yes	yes	no	yes	yes	yes	yes	yes	yes	9
Ubolsakka-Jones et al. (2019)	yes	yes	yes	yes	no	no	no	yes	yes	yes	yes	7
Srinivasan and Rajkumar, (2019)	yes	yes	yes	yes	no	no	no	yes	yes	yes	yes	7
Modesti et al. (2010)	yes	yes	yes	yes	no	no	no	yes	yes	yes	yes	7
Ubolsakka-Jones et al. (2017)	yes	yes	yes	yes	no	no	no	yes	yes	yes	yes	7
Adhana et al. (2013)	yes	no	no	yes	no	no	no	yes	yes	yes	yes	5
Total	20	18	19	20	3	3	5	20	20	20	20	7,4

### 3.2 Study characteristic

A total of 686 articles were retrieved from the three selected databases. After all the screening, 20 articles were included in this review. Of the 20 selected studies, most were conducted in Thailand (*n* = 7), while others were published in China (*n* = 3), India (*n* = 3), Malaysia (*n* = 1), Brazil (*n* = 2), Italy (*n* = 1), Taiwan (*n* = 1), Poland (*n* = 1), and United States of America (*n* = 1). The 19 selected articles were randomized controlled trial (RCTs), while 1 article was considered prospective observational. A total of 940 respondents involved in this research. All studies aim to find out whether breathing exercises can be beneficial for lowering blood pressure in hypertensive patients. 14 of the 20 studies were conducted on respondents with stage 1 and 2 essential hypertension, two studies were conducted on respondents with pre-hypertension, and four studies were conducted on respondents with isolated systolic hypertension (ISH). The age range of the respondents was 18–75 years (Table 4).

**TABLE 4:** Characteristics and outcomes of included studies.

No	Author	Title	Population characteristics	Study design	Sample	Age, gender	Outcome
1.	Jones et al. (2010)	An Inspiratory load enhances the antihypertensive effects of home-based training with slow deep breathing: a randomized trial	Inclusion criteria	RCT	30	35–65 years; male and female	- SBP
			- Essential hypertension Stage I or II				- DBP
			- good communication				- PP
			- independent ambulation				- MAP
			Exclusion criteria				
			- secondary hypertension				
			- respiratory disease				
			- diabetes mellitus				
			- cardiac, renal, or cerebrovascular disease				
			- dyslipidemia				
			- pregnancy within the last 6 months				
2.	Lin et al. (2012)	Heart Rate Variability Biofeedback Decreases	asymptomatic prehypertension	RCT	43	mean age: 22, 3 years, male and female	- HRV
		Blood Pressure in Prehypertensive Subjects by Improving Autonomic Function and Baroreflex					- BP
							- BRS
3.	de Barros et al. (2017)	Effects of long term device-guided slow breathing on sympathetic nervous activity in hypertensive patients: a randomized open-label clinical trial	The inclusions criteria were	RCT	20	- Both genders ≥18 years old,-	- sympathetic nerve activity (SNA) measured
			- with or without pharmacological treatment				- plasma catecholamines
			- mean 24-h BP by ABPM above the normal range				- BP
			- SBP ≥130 mmHg and/or DBP ≥80 mmHg				
			Exclusion criteria were				
			- in use of beta-blockers or centrally acting sympatholytic agents				
			- 3 or more antihypertensive drugs				
			- secondary hypertension, chronic respiratory disease, diabetes mellitus, a chronic renal disease defined as estimated glomerular filtration rate (eGFR) by the equation MDRD Study<60 ml/min, coronary artery disease, congestive heart failure				
			- pregnant women				
			- patients with a BMI >30 kg/m2				
4.	Wang et al. (2021)	Long-Term Effect of Device-Guided Slow	Essential hypertension patients	Prospective observational study	- 46	- 35–75 years	- BP
		Breathing on Blood Pressure Regulation and Chronic Inflammation in Patients with Essential	Inclusion criteria: patients who received medications and had good lifestyle control			- Male and female	- HRV
		Hypertension Using a Wearable ECG Device	Exclusion criteria				
			1. Recent medication titration related to BP or ANS function within 2 weeks				
			2. Recent major surgery or admission within 1 year				
			3. Had a history of anemia, asthma, thyroid dysfunction or autonomic neuropathy, or 4. Lived at high altitudes or had recently visited high mountainous areas for more than 1 week				
5.	Hering et al. (2013)	Effects of acute and long-term slow breathing exercise	- Men with newly diagnosed essential hypertension	RCT	- 28	- 37 ± 4.0 years, Male	- BP
		On muscle sympathetic nerve activity in untreated male patients with hypertension	- Office SBP between 140 and 160 mmHg and DBP lower than 95 mmHg				- HR
							- MSNA
6.	Wang et al. (2010)	Effect of Slow Abdominal Breathing Combined with Biofeedback on Blood Pressure and Heart Rate Variability in Prehypertension	prehypertension postmenopausal women	RCT	- 22	- ages 45–60 years	- BP
						Female	- HRV
7.	Mitsungnern et al. (2021)	The effect of pursed-lip breathing combined with number counting on blood pressure and heart rate in hypertensive urgency patients: A randomized controlled trial	Inclusion criteria: HT urgency	RCT	110	- aged 18–80 years old male ad female	BP
			Exclusion criteria				
			- cardiac arrhythmias, acute HF, acute coronary syndrome (ACS), acute stroke, acute respiratory failure				
			- alteration of consciousness				
			- pregnancy				
8.	Anderson et al. (2010)	Regular Slow Breathing exercises effects on Blood pressure and breathing patterns at rest	Prehypertension/Mild hypertension	RCT	40	53.4 ± 2.8	BP
			Inclusion criteria: mean systolic BP of the 10 measurements during the two sessions was >130 mmHg and <160 mmHg and mean diastolic BP < 100 mmHg				Breathing rate, tidal volume and minute ventilation
			Exclusion criteria				
			1. Had respiratory, cardiovascular, & renal disease				
			2. Were diabetes or the use of tobacco, steroids, hormone-replacement therapy, angiotensin II receptor blockers, angiotensin-converting enzyme inhibitors, b-blockers or, any other medications that would interfere with central nervous system activity				
9.	Sangthong et al. (2016)	Breathing Training for Older Patients with Controlled Isolated Systolic Hypertension	Inclusion criteria were mild to moderate ISH and older than 60 yr with constant medication for at least 1 month before the study	prospective randomized controlled trial	30	older than 60 yr male and female	BP
			Exclusion criteria				HR
			- regularly exercised				PP
			- had active cardiovascular disease, stroke, chronic renal failure, or chronic respiratory disease				
			- habitually taking supplements or herbal medicines that might affect blood pressure				
10.	Jones et al. (2015)	Slow Breathing Training Reduces Resting Blood Pressure and the	Inclusion criteria	RCT	30	aged 35–65 years	the SBP response to handgrip exercise
		Pressure Responses to Exercise	- essential hypertension, stage I-II				
			- an independent lifestyle				
			Exclusion criteria were				
			- blood pressure greater 180/110 mmHg or secondary hypertension				
			- respiratory disease, diabetes mellitus, heart, renal or cerebrovascular disease, dyslipidemia				
			- pregnancy within the last 6 months				
11.	Ping et al. (2018)	The impact of music guided deep breathing exercises on blood pressure control - A participant blinded randomized controlled study	stage 1 essential hypertension diagnosed at least 6 months before study entry with or without antihypertensive medications	RCT	87	62.6 ± 9.6 Male and female	BP
			Inclusion criteria				
			- should be stable on anti-hypertensive treatment for a minimum of 2 months before the study and - no change in medications during participation in the trial				
			Exclusion criteria				
			1. With impaired hearing, ischaemic heart disease, congestive heart failure, renal impairment				
			2. Diabetes mellitus of suboptimal control (HbA1C > 7 mmol/L)				
			3. Stroke within the previous 2 years				
			4. Major organ failure				
			5. Respiratory diseases				
			6. Resulting in dyspnoea at rest and those who were unable to operate a CD player or did not have access to a CD player				
12.	Li et al. (2018)	Effects of slow breathing rate on heart rate variability and arterial baroreflex sensitivity in essential hypertension	inclusion criteria	RCT	60	- 54.08 ± 5.18	- BP
			- Essential hypertension			- Male and female	- HRV
			- not taking any medication				- Arterial baroreflex sensitivity
			- non-smokers				
			- none was involved in competitive sports activities				
			Exclusion criteria				
			- ischemic heart disease, congestive heart failure, chronic atrial fibrillation, renal failure				
			- diabetes mellitus				
			- previous stroke				
			- major organ failure				
			- respiratory diseases, psychiatric disorders, and hearing impairment				
13.	Ublosakka-Jones et al. (2018)	Slow loaded breathing training improves blood pressure, lung capacity, and arm exercise endurance for older people with treated and stable isolated systolic hypertension	Inclusion criteria	RCT 8 weeks	66.4 ± 4.2	- 66.4 ± 4.2 male and female	- BP
			- with controlled mild to moderate ISH				- HR
			- being independently active and with good communication				- Maximal inspiratory pressure
			Exclusion criteria were				- Spirometry testing
			- secondary hypertension, use of beta-blockers				- Chest wall and abdominal expansion
			- heart or respiratory disease and arm exercise limited by pain				
14.	Kalaivani et al. (2019)	Effect of alternate nostril breathing exercise on blood pressure, heart rate, and rate pressure product among patients with hypertension in JIPMER, Puduccherry	- Inclusion criteria	RCT	- 170	- 51–60 years	- BP
			- Men and women 30 sd 60 years old			- Male ang female	- HR
			- patients diagnosed with mild and moderate hypertension taking antihypertensive medication				
			- Exclusion criteria: have a previous experience of yoga and patients who were chronic smokers				
15.	Oneda et al. (2010)	Sympathetic nerve activity is decreased during device-guided slow breathing	- Inclusion criteria	RCT	- 31	- 51 ± 9 years	- BP
			- Mild hypertesive			- Male and female	- HR
			- Non-diabetic, non-obese				- Respiratory Rate (RR)
			- receiving stable treatment for hypertension, with no changes 1 month before the experimental session				- MSNA
			- Exclusion criteria: Receive sympatholitics				
16.	Ubolsakka-Jones et al. (2019)	The effects of slow loaded breathing training on exercise blood pressure in isolated systolic hypertension	Inclusion criteria	RCT	- 22	- 67 ± 6 years	- Resting BP (the end of 8 weeks training period, at 12 and 16 weeks after training)
			- age 60–80 years with stable controlled mild to moderate ISH, (average resting sBP >140 mmHg and dBP <90 mmHg)			- Male and female	- Resting HR
			- good communication, and independent living				- BP and HR exercise
			Exclusion criteria				
			- secondary hypertension				
			- use of beta-blockers				
			- heart disease, respiratory disease, neuromuscular disease, arm exercise limited by pain				
17.	Srinivasan and Rajkumar, (2019)	Effects of slow breathing on blood pressure and end tidal carbon dioxide in hypertension: randomised controlled trial	Inclusion criteria: pre- and stage I IHT (120–159 mmHg)/(80–99 mmHg), age range: 30–60 years and both the genders	RCT	- 40	45.10 ± 8.25 male and female	- BP
							- End tidal CO2
							- HR
18.	Modesti et al. (2010)	Psychological predictors of the antihypertensive effects of music guided slow breathing	Inclusion criteria	RCT	- 29	- 40–75 years	- BP
			- outpatients aged 40–75 years with essential hypertension, untreated or constantly treated with the same doses of antihypertensive drugs for at least 3 months prior to the study				- Quality of life
			Exclusion criteria				- Psychological subscale
			- chronic atrial fibrillation, angina, heart failure, cerebrovascular disease, diabetes mellitus, renal failure, asthma, chronic respiratory disease, pregnancy, neoplasia and altered night-time sleep because of shift work				
19.	Ubolsakka-Jones et al. (2017)	The effect of slow-loaded breathing training on the blood pressure response to handgrip exercise in patients with isolated systolic hypertension	Inclusion criteria	prospective randomized trial	- 30	- 60–70 years	- Resting BP and HR
			- mild-to-moderate ISH			- Male and female	- BP and HR followed by a static handgrip exercise test
			- over 60 years				
			- constant medication for at least 1 month prior to the study				
			Exclusion criteria				
			- regularly exercised had active cardiovascular disease, stroke, chronic renal failure or chronic respiratory disease				
20.	Adhana et al. (2013)	The influence of the 2:1 yogic breathing technique on essential hypertension	Inclusion criteria	RCT	- 30	- ages of 20–50 years	- EMG
			- Males and females between ages of 20–50 years			- male dan female	- GSR (Galvanic skin response)
			- newly diagnosed of having essential hypertension in prehypertensive stage and stage 1				- FTT (Finger tip temperature)
			- Not taking treatment				- HR
			- No past history of any chronic illness like chronic renal failure, uncontrolled diabetes mellitus				- RR
			Exclusion criteria				- BP
			- Significant co-morbidity like angina				
			- uncontrolled diabetes mellitus, chronic renal failure, stroke, obesity				

ABPM, Ambulatory blood pressure monitoring; ACS, Acute coronary syndrome; ANS, Autonomic nervous system; BMI, Body mass index; BP, Blood pressure; BRS, Baroreflex sensitivity; DBP, Diastolic blood pressure; FTT, Finger tip temperature; GSR, Galvanic skin response; HF, Hheart failure; HR, Heart rate; HRV, Heart rate variability; HT, Hypertension; ISH, Isolated systolic hypertension; MAP Mean arterial pressure; MSNA, Muscle sympathetic nerve activity; PP, Pulse pressure; RCT, Randomized control trial; RR, Respiratory rate; SBP, Systolic blood pressure; SNA, Sympathetic nerve activity.

Resting blood pressure and heart rate was utilized as measures in almost all investigations. Blood pressure and heart rate responses to handgrip exercises were measured in two investigations, while ambulatory blood pressure was measured in two others. Autonomic nerve dysfunction is one of the causes of hypertension. There are five publications in this study that use MSNA (Muscle sympathetic nerve activity) (de Barros et al., 2017) (Hering et al., 2013) (Oneda et al., 2010), HRV (Heart Rate Variability) (Wang et al., 2010) (Wang et al., 2021), and three studies that use baroreflex sensitivity parameter to assess autonomic nerve status (Wang et al., 2021) (Lin et al., 2012) (Li et al., 2018). Blood inflammatory indicators, such as tumor necrosis factor-alpha (TNF-α), interleukin-6, interleukin-1 receptor agonist, and C-reactive protein (Wang et al., 2021), lung capacity, chest and abdominal expansion (Fonkoue et al., 2018), and plasma catecholamines, were also included in this review (de Barros et al., 2017).

### 3.3 Type of breathing exercise

Breathing exercises are techniques that are easy to do and do not require a lot of muscle work, so all ages can do them. 16 of 20 studies used slow breathing to lower blood pressure with a frequency that varied between 4 and 10 breaths per minute. Six studies used slow-loaded breathing, and six studies used device-guided slow breathing. Loaded breathing is done using a Water Pressure Threshold Bottle by providing an inspiration load of 20 cm H_2_O (Jones et al., 2010) (Jones et al., 2015) or 18 cmH_2_O (Sangthong et al., 2016). Meanwhile, Chulee Ublosakka uses a BreatheMAX device with a 25% MIP load (Ublosakka-Jones et al., 2018) (Ubolsakka-Jones et al., 2019). To guide the respondent to do slow breathing, a guided slow breathing device is used. Wang used a wearable ECG wristband (MiCor A100, MiTAC Corp., Taiwan) (C. Wang et al., 2021), while Anderson used a device that included a microcomputer connected to a band worn around the torso and a set of earphones (Anderson et al., 2010) (de Barros et al., 2017). One study used pursed lip breathing (PLB) and the other used alternate nostril breathing, pranayama, and yoga (Table 5).

**TABLE 5:** Type, rate, dan frequency of breathing training.

No	Author	Type of breathing training	Respiratory rate	Frequency and duration	Change in BP
1.	Jones et al. (2010)	slow deep breathing at home, either unloaded or loaded breathing	6 cycles/min	30 min, twice a day, every day for 8 weeks	- unloaded breathing: 7.0 mmHg (SBP) and 13.5 mmHg (DBP)
					- loaded breathing: 18.8 mmHg (SBP) and 8.6 mmHg (DBP)
2.	Lin et al. (2012)	heart rate variability–biofeedback (HRV-BF) slow abdominal breathing	6 cycles/min	20-min period twice a day for 5 weeks	HRV-BF
					SBP decreased by 12,8 mmHg
					DBP decreased by 7,4 mmHg
					SAB
					SBP decreased by 7,7 mmHg
					DBP decresed by 4,4 mmHg
3.	de Barros et al. (2017)	Device-guided slow breathing	≤10 breaths/min	daily for 15 min for 8 weeks	no changes
4.	Wang et al. (2021)	device-guided slow breathing	6 cycles/minute	3 min, five times per day over the 3 months	no changes in SBP
5.	Hering et al. (2013)	SLOWB exercise using device guided breathing	10 breaths/min	15-min daily sessions over 8 weeks	no changes
6.	Wang et al. (2010)	slow abdominal breathing combined with frontal EMG biofeedback training and daily home practice	6 cycles/min	10 sessions of treatment once every 3 days. Each session lasted 25 min twice every day at home, with each period lasting 20 min	SBP decreased by 8.4 mmHg and the DBP by 3.9 mmHg
7.	Mitsungnern et al. (2021)	pursed-lip breathing and number counting	8.5 cycles/min	15 min (min) of each hour for 3 h	SBP: 28.2 mmHg DBP: 17.1 mmHg
8.	Anderson et al. (2010)	device-guided slowbreathing (DGB) exercise	≤10 breaths/min	daily 15-min sessions for 4 weeks	
9.	Sangthong et al. (2016)	loaded breathing (18 cm H2O) and unloaded breathing	six breaths per min	30 min every day for 8 weeks	SBP was reduced by 18 ± 7 (loaded group) and 11 ± 4 mmHg (unloaded groups)
10.	Jones et al. (2015)	slow breathing	6 cycles/min	30 min, twice a day, and every day for 8 weeks	sBP response to handgrip exercise after training was reduced by 10 mmHg and HR by 5 bpm
		Unloaded and loaded (20 cm H2O)			
11.	Ping et al. (2018)	music guided, slow and deep breathing	5 cycles/min	15 min per day for 8 weeks	Music guided
					10.5 mmHg
					Music + BE: 8.3 mmHg
12	Li et al. (2018)	slow breathing	8 and 16 breaths per minute	5 min for 2 periode	Resting SBP: from 150.36 ± 12.9 to 146.63 ± 11.12 (8 b/m) dan from 150.36 ± 12.9 to 152.57 ± 10.35 (16 b/m)
13.	Ublosakka-Jones et al. (2018)	Slow loaded breathing training (SLB) group with a load of 25% MIP using a BreatheMAX device	6 breaths per minute	total of 60 breaths a day, every day for 8 weeks	Resting SBP decreased by 20 mmHg for SLB and by 5 mmHg for CON.
					DBP decreased significantly for SLB (9 mmHg)
14.	Kalaivani et al. (2019)	alternate nostril breathing exercise	Not mention	two times a day (10 min duration of exercise each time) for 5 days	Decreased SBP by 54,22 mmHg
					Decreased DBP by 6,47 mmHg
					Decreased hert rate by 3,41 bpm
15.	de Barros et al. (2017)	device-guided slow breathing (breathe with interactive music (BIM))	<10 breaths min-1	15 min	SBP: decreased by 6 mmHg (BIM) and 4 mmHg (CG)
					DBP: decresed by 4 mmHg (BIM) and 3 mmHg (CG)
					MSNA: reduced by 8bursts/min (BIM)
16.	Ubolsakka-Jones et al. (2019)	slow loaded breathing (SLB: 25% maximum inspiratory pressure	6 breaths per minute	60 breaths a day, every day for 8 weeks	Home SBP decreased by 22 mmHg, DBP by 9 mmHg and HR by 12 bpm
17.	Srinivasan & Rajkumar, (2019)	Slow breathing training	6 breaths/minute	30 min	SBP reduced by 12.3 mmHg
					DBP reduced by 3.9 mmHg
18.	Modesti et al. (2010)	Buteyko and pranayama breathing technique (voluntary music-guided slow breathing)	4–6 breaths/min	30-min daily for 6 onths	Office SBP decreased by 7,4 mmHg and DBP decreased by 3,9 mmHg
19.	Ubolsakka-Jones et al. (2017)	Slow loaded breathing training (loaded breathing Training)	6 breaths/min	30 min a day every day for 8 weeks	resting SBP decreased by 10.6 mmHg
					the SBP at the end of exercise was reduced by 12.6 mmHg
20.	Adhana et al. (2013)	2:1 yogic breathing technique	5 breaths/min	twice a day for 5–7 min for 3 months	SBP decreased by 12 mmHg and DBP by 7 mmHg

BIM, Breathe with interactive music; CG, Control group; CON, Deep breathing control; DBP, Diastolic blood pressure; DGB, Device-guided slow breathing; EMG, Electromyographic; HR, Heart rate; HRV-BF, Heart rate variability biofeedback; MSNA, Muscle sympathetic nerve activity; SAB Slow abdominal breathing; SBP, Systolic blood pressure; SLOWB, Slow breathing; SLB, Slow loaded breathing.

### 3.4 Frequency and duration

Most of the studies selected in this scoping review applied daily breathing exercises, 10–60 min a day. Breathing exercises were used in some research for 15 min daily (7 studies), 30 min daily (4 studies), and 60 min daily (2 studies). Six studies were performed two times per day, the rest did the exercises once a day. There are 2 studies that have done breathing exercises 60 times a day with a frequency of 6 breaths per minute. The minimum duration was 3 min for 1 session, which is done 5 times a day so the total training time was 15 min per day. The effect of breathing exercises was seen in the duration of 12 weeks (2 studies), 8 weeks (12 studies), 6 months, 5 weeks, 4 weeks, and 5 days each in 1 study. There were 2 studies that looked at the direct effect of breathing exercises.

### 3.5 Effects of breathing exercises on blood pressure parameters

The effects of breathing exercise on cardiovascular condition, autonomic nerve activity, blood inflammatory biomarkers, and lung health were studied in this study. Only three research reported no change in blood pressure after receiving breathing training treatment, despite the fact that 17 studies reported a decrease in blood pressure, both systolic and diastolic. The systolic blood pressure declined by 4–54.22 mmHg, while the diastolic blood pressure dropped by 3–17 mmHg.

The autonomic nervous activity was determined in 5 of the 20 papers. MNSA was the subject of three studies, but only one of the recommended revisions. Sympathetic nerve activity reduces as people breathe slowly. Muscle sympathetic activity decreases during the fifth and 10th minutes of BIM use. At the time, the breathing frequency was already around ten breaths per minute, implying that sympathetic activity is linked to breathing frequency (Oneda et al., 2010).

In contrast to the Oneda study, de Barros et al. (2017) found that long-term device-guided slowbreathing (DGB) did not affect blood pressure, catecholamine levels, or MSNA in hypertensive individuals. Long-term slow breathing (SLOWB) training lowers office BP and HR, but not 24-h ambulatory BP and HR; SLOWB selectively attenuates cardiovascular effects of mental, but not physical stressors in this cohort of patients with untreated essential hypertension; and SLOWB reduces MSNA during acute device-guided lowering of breathing frequency, but not by long-term SLOWB home exercises (Hering et al., 2013).

According to a study, TNF alpha, IL 6, IL 1 receptor agonist, and C reactive protein levels are lower. TNF-α decreased significantly throughout the 3-month training period: the difference was significant after 1 month of DGB exercise training (*p* 0.05), and it continued to diminish after 3 months of training (*p* 0.05). TNF-α decrease levels did not change significantly between participants with and without comorbidities (all *p* > 0.05). Other biomarkers such as IL-6, IL-1ra, galectin-3, and CRP, on the other hand, did not demonstrate any significant alterations during the study period.

According to an article, the SLB group showed significant increases in chest and abdominal expansion, most likely due to their enhanced inspiratory muscle strength. With SLB, increased chest and abdominal expansion was linked to considerably bigger slow vital capacity (SVC) and inspiratory capacity (IC). The SLB has a higher tidal volume due to the lower breathing rate. Meanwhile, one study found that after being given Device-guided slow breathing, there was no change in plasma catecholamines (de Barros et al., 2017).

## 4 Discussion

An imbalance of the autonomic nervous system in the form of increased sympathetic activity and decreased parasympathetic activity has a major role in the etiology of hypertension (Howorka et al., 2013). Another factor that contributes to the incidence of hypertension is impaired baroreflex sensitivity.

According to research, slow breathing lowers sympathetic tone and raises parasympathetic tone. This could be partially mediated by alterations in intrathoracic pressure (Toska and Eriksen, 1993; Triedman and Saul, 1994), stimulation of arterial and cardiopulmonary baroreceptors and pulmonary afferent stretch receptors or by central interactions between respiratory and cardiovascular centers in brainstem modulation of vagal activity during breathing (Eckberg and Karemaker, 2009).

Slow breathing is defined as a respiratory rate of fewer than 10 breaths per minute with prolonged, rhythmic, slow, and deep expiratory periods. Slow and deep breathing can have a relaxing effect, through changes in the body’s biochemistry, such as increasing endorphins (substances that cause relaxation), lowering adrenaline, and lowering blood acidity (Pickering et al., 2005), increasing baroreflex sensitivity, and lowering blood pressure (Joseph et al., 2005) This breathing technique increases the length of the diaphragm contraction, minimizes the respiratory rate, and deepens the volume of inspiration and expiration, thus maximizing the amount of oxygen entering the bloodstream. Deep breathing exercises have been shown in numerous trials to be beneficial for hypertension patients (Joseph et al., 2005; Ma et al., 2017; Srinivasan and Rajkumar, 2019). In hypertensive patients, slow and deep breathing exercises over a period of weeks can significantly lower SBP and DBP (Elliot et al., 2004; Mourya et al., 2009), enhance baroreflex sensitivity, and significantly boost HRV in both prehypertensive and hypertensive people.

Reduced SBP and enhanced psychological well-being are linked to elevated HRV (Elliot et al., 2004; Wang et al., 2010). In hypertensive people, slow deep breathing for 2 minutes can lower SBP and DBP by 8.6 and 4.9 mmHg, respectively (Joseph et al., 2005). The lung stretch reflex, which prevents sympathetic outflow, can be activated during slow, deep breathing by having extended inspiratory phases (4 s) and high lung volumes (Seals et al., 1993). In addition, slow breathing also increases the interaction between respiratory neurons with the heart and autonomic centers in the brainstem. Respiratory sinus arrhythmias are much more pronounced during slow breathing at about six breaths per minute and are also greater with large lung volumes (Brown et al., 1993).

Additionally, there may be a close connection between the respiratory center, where voluntary control is present, and the autonomic nervous system, as evidenced by the fluctuating activity of the muscle sympathetic nerve and baroreceptor sensitivity during the respiratory cycle (Eckberg, 1980; St. Croix et al., 1999; Eckberg, 2003; Shantsila et al., 2015). Therefore, intentional slow breathing during exercise has the potential to change the neural pathways that control both resting blood pressure and how the body reacts to physical exertion. Numerous studies have demonstrated that six- to ten-breaths-per-minute deep diaphragmatic breathing exercises can increase arterial dilatation by stimulating heart-lung mechanoreceptors while reducing sympathetic nerve activity and chemoreflex activation. It raises parasympathetic activity and baroreflex sensitivity in hypertensive patients, lowering SBP and DBP (Elliot et al., 2004; Joseph et al., 2005; Mourya et al., 2009; Wang et al., 2010; Srinivasan and Rajkumar, 2019).

By stimulating stretch receptors in the aortic arch and carotid sinuses, the baroreflex is activated, which is one of the main mechanisms relating to the favorable cardiovascular effects of deep breathing (Heusser et al., 2010). By activating arterial baroreceptors, elevated afferent neural discharge to central-neural-autonomic areas causes increased parasympathetic efferent activity through the vagus nerve to the sinoatrial (SA) node, which in turn causes decreases in HR (Kougias et al., 2010). Then, it’s probable that changes in intrathoracic pressure brought on by the thorax expanding cause changes in the venous filling, stroke volume, cardiac output, and peripheral blood flow (the respiratory pump), which in turn causes a brief increase in blood pressure (Russo et al., 2017). The parasympathetic nervous system is then activated, which causes a drop in heart rate. Studying the impact of breathing patterns on baroreflex function is crucial because it reveals that slow breathing at 0.1 Hz (with an inspiration/expiration ratio of 1) enhances baroreflex gain (Wang et al., 2013).

### 4.1 Research gaps and implications for future research

We found many actionable research needs as a result of our study. First, because hypertension is linked to stress and anxiety, more research is needed to determine the effects of breathing exercises in lowering stress and anxiety, which can lead to reduced blood pressure. Second, only one journal in this review measured plasma catecholamines, whereas an increase in blood pressure or hypertension is frequently accompanied by metabolic changes such as impaired glucose tolerance, hyperinsulinemia, hyperlipidemia, obesity, humeral changes such as increased renin activity, plasma, catecholamines, and aldosterone, and hemodynamic changes such as left ventricular hypertrophy and impaired diastolic function, as well as humeral changes such as increased renin activity, More research is needed to establish the impact of breathing exercises on metabolic parameters in the body.

Because the time spent conducting breathing exercises varied widely in this analysis, more research is needed to discover the minimal duration and intensity required to produce advantages for hypertension patients in decreasing blood pressure and many cardiovascular parameters. Finally, a study on how long breathing exercises can help hypertension patients should be conducted.

It is noteworthy that the included studies demonstrate a homogenous breathing exercise technique, which enables us to draw more conclusive findings. We also conducted quality appraisals on all included studies to improve the trustworthiness of our review. However, even though we did not limit the geographical region in our article search, the retrieved studies mainly originated from Asia countries. This might limit the generalization of the finding from this review.

## 5 Conclusion

Slow deep breathing can be a non-pharmacological alternative therapy for people with hypertension in addition to lifestyle modification. Slow deep breathing is easy for people of all ages and does not have to be expensive. This research gathered 20 studies on the benefits of breathing exercises in hypertension patients. Breathing exercise lowers blood pressure and pulse during and after exercise, as well as MSNA, but it has no effect on plasma catecholamines. In one trial, inflammatory biomarkers (TNF alpha, IL 6, IL 1 receptor agonist, and C reactive protein) decreased, but CRP remained unchanged. Enhanced chest and abdominal expansion, as well as increased inspiratory muscular strength, were also obtained. One There were no significant changes in plasma catecholamines, according to the study. There are still some questions to be answered about the frequency and intensity of breathing exercises that are useful for hypertension patients and how long the effects last.

This review mapped the outcomes of breathing exercises, one of the non-pharmacological approaches to managing hypertension. Almost all included studies demonstrate that this affordable approach leads to positive effects. This finding can be the basis of the breathing exercises implemented by healthcare providers for patients with hypertension.

## Data Availability

The raw data supporting the conclusions of this article will be made available by the authors, without undue reservation.
